# Thermo-Mechanical and Fatigue Behavior of 3D-Printed PA12 CF15 for Engineering Application

**DOI:** 10.3390/polym18050563

**Published:** 2026-02-26

**Authors:** Justas Ciganas, Tomas Kalinauskis, Urte Cigane

**Affiliations:** Siauliai State Higher Education Institution, Ausros al. 40, LT 76241 Siauliai, Lithuania; j.ciganas@svako.lt (J.C.); t.kalinauskis@svako.lt (T.K.)

**Keywords:** PA12 CF15, quasi-static tensile, fatigue, dynamic mechanical analysis (DMA), 3D printing, engineering application, finite element analysis

## Abstract

This study presents a detailed experimental investigation of the mechanical, fatigue, and dynamic properties of a 3D-printed PA12 CF15 composite at different temperatures. The mechanical properties determined in the temperature range from 23 °C to 120 °C were later implemented in numerical simulations to evaluate the suitability of the material for thermo-mechanical loading conditions. Quasi-static tensile test results revealed a decrease in elastic modulus, yield strength, and ultimate tensile strength with increasing temperature. Fatigue testing demonstrated that increasing load levels lead to reduced durability and a lower maximum number of cycles to failure. Furthermore, elevated testing temperatures caused the composite to exhibit more pronounced plastic behavior, resulting in temperature-dependent fatigue performance. SEM analysis indicated that higher temperatures increase the plasticity of the composite, thereby reducing the reinforcing effect of carbon fibers. The mechanical characteristics obtained experimentally were incorporated into a finite element model, allowing a preliminary assessment of the feasibility of manufacturing an intake manifold from PA12 CF15 using additive manufacturing technology. The results of this study provide valuable data for the design and analysis of dynamically and thermally loaded engineering components produced from PA12 CF15 composites.

## 1. Introduction

Polymeric materials have become an integral part of modern technology due to their versatility and wide range of functional capabilities [1]. In addition, in the last decade, the widespread adoption of polymer composite materials in numerous industrial and scientific fields has been strongly driven by the incorporation of various micro and nanoparticles, which significantly enhance the properties of the matrix and reinforcing phases in hybrid composite systems [2]. Polymers and polymeric materials can be used in various fields, from sensors [3,4,5] to biomedical applications [6,7], packaging [8,9] and water purification technologies [10,11]. Due to their favorable combination of mechanical properties and environmental resistance, polymer composites are also particularly valued in industries where high dynamic durability is required, such as automotive [12,13,14,15] and aerospace [16,17].

Polymer composite materials are increasingly used in the automotive industry to achieve significant reductions in vehicle weight, thereby improving fuel efficiency and reducing gas emissions. Incorporation of fiber-reinforced polymer matrix composites facilitates a marked reduction in component mass while ensuring mechanical performance comparable to or exceeding that of conventional metallic materials. The automotive industry is increasingly focused on vehicle light weight as a key strategy to reduce energy consumption and mitigate environmental impacts [18]. Composite materials, particularly fiber-reinforced polymers with high strength-to-weight ratios, offer significant potential to reduce vehicle mass while maintaining high performance and improving aerodynamic efficiency. Consequently, both industry and academia are extensively investigating the adoption of such materials as a means to reduce fuel consumption and reduce harmful emissions in conventional and electric vehicles [19].

In engineering applications [20,21], additive manufacturing (AM) techniques, particularly extrusion-based methods such as Fused Filament Fabrication (FFF), have significantly expanded the potential of polymer components. As composite materials improve, three-dimensional (3D) printing is becoming more widely used, although the resulting structure is not homogeneous, the mechanical properties are becoming increasingly suitable for different applications [22]. FFF technology enables rapid prototyping, low-volume production, and the fabrication of geometrically complex structures without molds, making it increasingly attractive for the automotive sector. 3D printing technologies allow for the creation of unique intake system geometries in automobiles, reducing weight and improving performance, and other automotive components have also been manufactured using the FFF process [23].

As the range of materials available for 3D printing expands from polymers such as PLA to metals including aluminum and titanium alloys, their range of applications in various engineering and industrial fields continues to increase [24]. With the adoption of polyamide 12 (PA12) in 3D printing, it has become widely used in various engineering fields due to its favorable balance of mechanical strength, thermal stability, chemical resistance and good processability, particularly for automotive and industrial components [25]. Due to its good mechanical properties, the PA12 polymer has been start modified with different fibers to achieve improved stiffness, reduced thermal expansion, and greater dimensional stability compared to pure PA12 [26,27]. Carbon fiber is among the strongest reinforcing fibers and, when incorporated into composite materials, can significantly enhance mechanical properties and thermal resistance [28], which are critical requirements for under-the-hood automotive components. However, despite these advantages, the mechanical behavior of FFF-printed PA12-CF composites strongly depends on printing parameters, layer adhesion quality, fiber orientation, and the inherent anisotropy of the extrusion-based process [29]. Therefore, extensive research is required on such polymer composites, as their mechanical and thermal behavior can vary significantly depending on the printing conditions, and the presence of additives at different concentrations can substantially alter the resulting polymer properties [30].

In this context, understanding the performance of polymer composites under real service conditions becomes particularly important. Automotive components are subjected to a wide range of loading conditions depending on their application, including combined mechanical and thermal loads. In particular, automotive air-intake components operate in thermally and mechanically demanding environments, where materials must maintain structural integrity and functional performance over extended service periods. These parts are exposed to elevated temperatures, fluctuating temperature cycles, continuous vibrations, airflow pulsations, and long-term mechanical loading [31]. While injection-molded polyamides are well established in these applications, additively manufactured carbon fiber–reinforced PA12 may exhibit different mechanical characteristics due to fiber orientation, porosity, and the layer-by-layer deposition characteristics inherent in FFF. Consequently, understanding how temperature influences stiffness, strength, damping behavior, and fatigue life is essential to assess the suitability of FFF-printed PA12-CF materials in real under-hood conditions. The air-intake component was selected due to its uniqueness and functional importance, as modifications to its geometry can directly influence the overall performance characteristics of the vehicle. The mechanical loads acting on the component arise primarily from the mass of the attached throttle body and other auxiliary components. Due to continuous engine-induced vibrations, the attached mass behaves similarly to a pendulum, generating cyclic mechanical loads and contributing to combined thermal and fatigue load conditions during operation. Therefore, the aim of this study is to investigate the thermo-mechanical performance of PA12 reinforced with 15 wt.% short carbon fibers (PA12 CF15) produced using FFF technology. The research includes quasi-static tensile, fatigue tests, and dynamic mechanical analysis (DMA) conducted under controlled thermal conditions. DMA provides an efficient characterization of viscoelastic properties over a wide range of temperatures and strain rates. The experimental data obtained are further used in theoretical mathematical calculations to obtain the geometry response to operating temperatures and mechanical loads. Additionally, SEM analysis was performed to better understand fatigue formation. The obtained results provide insights into the material’s behavior under combined thermal and mechanical loading and help determine its potential for use in functional air-intake system components.

This article is distinguished by its novelty, as it presents a systematic thermomechanical and fatigue analysis of an FFF-printed PA12 CF15 composite. The study investigates the mechanical behavior of the composite in a temperature range of 23 °C to 120 °C, demonstrating the influence of temperature on stiffness degradation, damping behavior, and fatigue lifetime. The obtained experimental results can be used as primary input data for subsequent computational analyses of structural components manufactured from carbon fiber-reinforced PA12 CF15 composite material. Moreover, the present work also includes a case study in which experimental thermomechanical and fatigue data are utilized to predict the fatigue life of components to prevent premature failure. This comprehensive analysis extends the application potential of FFF technology in the automotive industry and other engineering fields where components are exposed to elevated operating temperatures and vibrational loading.

## 2. Materials and Methods

### 2.1. Materials

This study investigates the mechanical and thermal properties of the carbon fiber-reinforced polymer PA12 CF15. In the research, a nylon-based filament manufactured by “Fiberlogy” (Fiberlab S.A., Brzezie, Poland) was used, supplied on a 0.5 kg spool with a filament diameter of 1.75 mm. The basic properties provided by the manufacturer are summarized in Table 1.

Using FFF technology, the specimens were manufactured with a 3D printer “Creality K1 MAX” (Creality, Shenzhen, China). Since the printing parameters play a critical role in this type of study, the printing parameters were selected according to the manufacturer’s recommendations. The printing parameters of the specimens are summarized in Table 2.

**Figure 1:**
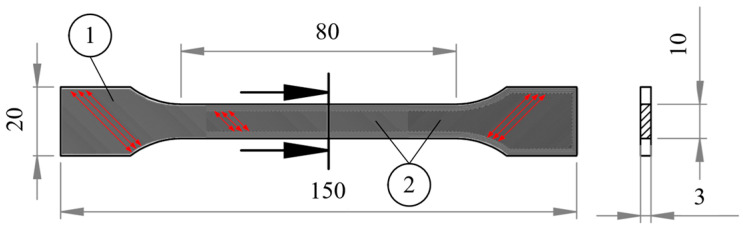
The geometry of the specimen: 1—top surface; 2—internal layer orientation of the 3D-printed specimen (the raster angle of 45° is indicated by red arrows).

The geometry of the specimen was selected according to the ISO 527 standard [33]. The total length of the specimen was 150 mm, with a gauge length of 80 mm. The specimen had a thickness of 3 mm, a width of 10 mm in the narrowest section, and 20 mm in the widest section. The thickness of the printing layer was chosen to be 0.2 mm and the raster angle was 45°/−45°. Each specimen was printed horizontally on the heated bed with 100% linear infill, ensuring consistent layer deposition. The geometry and raster angle of the specimen is shown in Figure 1.

Due to the characteristics of the additive manufacturing process, the produced specimens exhibited anisotropic mechanical behavior. However, the ISO 527 standard [33] assumes the homogeneity of the material. Therefore, it was necessary to define the printing parameters based on the findings reported in previous studies [36,37,38,39], since interlayer adhesion—and consequently mechanical performance—depends on these parameters [40]. Therefore, the exact printing parameters are given in Table 2.

Although PA12 has relatively low moisture absorption compared to other polyam-ides [36,41] or reinforced with carbon fibers [42], the PA polymer is hygroscopic and humidity can affect its mechanical properties. Therefore, before printing, the filaments were dried in a convection oven at 80 °C for 12 h. After the specimens were printed with the 3D printer, they were kept in a thermal chamber for a stabilization period to ensure a uniform temperature distribution throughout the specimens. The temperature of the thermal chamber was selected at the test temperatures and the specimens were kept for 30 min before the beginning of the experiment [43]. Although the specimen was dried, the moisture content of the material/specimen was not checked after drying and before testing, so it is worth noting that the moisture conditioning of PA12 CF15 may influence mechanical properties. The quasi-static tensile, fatigue, and DMA tests were performed under controlled climatic conditions with constant ambient temperature and constant humidity.

### 2.2. Methods

Quasi-static tensile tests were performed to determine the elastic modulus, yield strength, and other mechanical properties values for further use in modeling and fatigue tests. For high-temperature tensile testing, the ISO 527 standard [33] was used as in other similar tests [44]. During the experiment, three tensile tests were conducted for each temperature condition to ensure the reliability and repeatability of the results. The specimens were tested at a load rate of 1 mm/min. The investigated temperature range was selected from 23 °C to 120 °C. The engineering strain was determined from the crosshead displacement of the testing machine. Thus, the strain calculations were based on the crosshead displacement data. This methodology assumes that deformation is uniformly distributed over the entire 110 mm grip separation distance, which encompasses the 80 mm gauge section as well as portions of the transition regions. Consequently, the calculated elastic modulus should be regarded as an apparent modulus, as it inherently includes the influence of machine compliance.

Fatigue tests were performed to evaluate the fatigue behavior and durability of the material under cyclic loading conditions. For high-temperature fatigue testing, the ISO 13003 standard [45] was used. During the experiment, three fatigue tests were performed for each loading condition to ensure repeatability and statistical reliability of the results. The tests were carried out under force-controlled cyclic loading with a sinusoidal waveform. The fatigue tests were performed under stress control at a load ratio *R* = 0.1. The loading frequency was set to 2 Hz to avoid excessive self-heating of the specimens. The maximum amplitude stress for 23 °C was selected at 53.3 MPa and for 120 °C was selected at 20 MPa. The fatigue tests were performed until the specimen failed or until a predefined number of cycles was reached. The investigated temperature range was selected from 23 °C to 120 °C.

DMA was performed to determine the viscoelastic properties of the material, including the storage modulus (*E*′), the loss modulus (*E*″) and the damping factor (*tan δ*). The specimens were tested in tensile mode using the ISO 6721 standard [46]. A sinusoidal dynamic load was applied while maintaining the deformation amplitude within the linear viscoelastic region. The tests were carried out in multi-frequency strain-controlled mode: the temperature was increased from 23 °C to 120 °C and the heating rate was 1 °C/min. The testing environment was the air. At least 10 specimens were tested to ensure repeatability.

**Figure 2:**
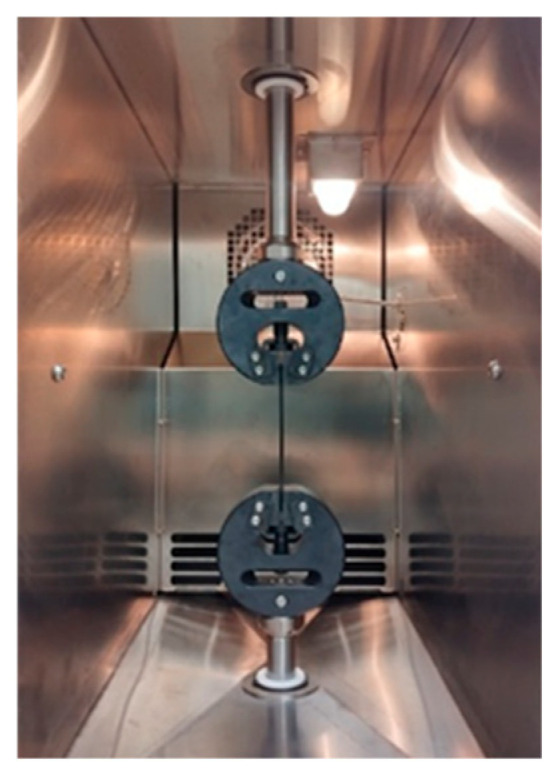
Specimen in universal testing machine grips in the heating chamber.

Quasi-static, fatigue and DMA tests were performed using the universal testing machine “Step-Lab UD08” (STEP Engineering, Resana, Italy) equipped with an integrated thermal chamber (STEP Engineering, Resana, Italy). The test temperature was maintained for a specified time according to the ISO 6721 standard [46]. The specimen in universal testing machine grips is shown in Figure 2.

Scanning electron microscopy (SEM) was used to investigate the surface morphology and fracture characteristics of the specimens after the tensile and fatigue tests. The analyses were performed to evaluate microstructural features such as surface defects, crack initiation sites, and failure mechanisms of the material. SEM observations were performed using a “FlexSEM 1000 II” microscope (Hitachi High-Tech, Tokyo, Japan) operating under high vacuum conditions.

## 3. Results and Discussions

### 3.1. Quasi-Static Tensile Tests

**Figure 3:**
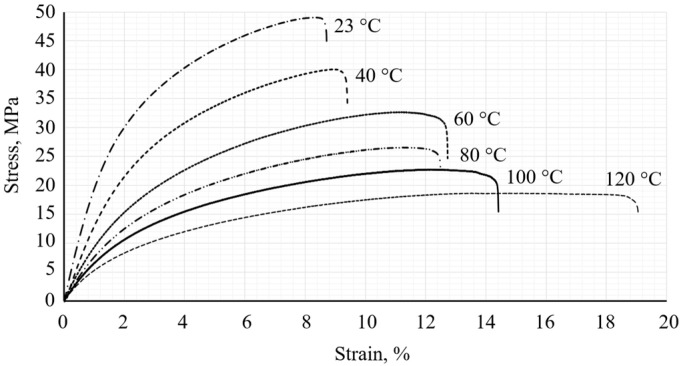
Stress–strain curves for PA12 CF15 specimens.

Quasi-static tensile tests conducted at different temperatures enabled the determination of the stress–strain curves, which are presented in Figure 3. Three main stages of the deformation process recorded during the experiment: elastic deformation, nonlinear deformation, and softening stage.

It should be emphasized that the specimens investigated in this study were manufactured using a specific set of printing parameters. The mechanical properties of polymer-based 3D-printed models may vary significantly when these parameters are modified. For this reason, numerous studies have investigated the influence of printing parameters on the mechanical properties of additively manufactured materials [47]. Such variations may be associated with changes in molecular arrangement or, in the case of fiber-reinforced polymers, with the orientation of carbon fibers. A dedicated experimental study would be required to systematically evaluate the influence of printing parameters on the mechanical behavior of the materials.

Based on the obtained results in this study, the main mechanical parameters, including elastic modulus, yield strength, ultimate tensile strength, and elongation at break, were calculated. A summary of these parameters is shown in Table 3.

The results presented in Table 3 show that elastic modulus, yield strength, and ultimate tensile strength decrease with increasing temperature, which is typical for thermoplastic materials. The decrease in elastic modulus, yield strength, and ultimate tensile strength indicates that the stiffness of the material decreases and the material becomes more deformable. This behavior can be attributed to the increased mobility of the polymer chains at higher temperatures, and the carbon fiber reinforcement can no longer fully compensate for the softening effect. Similar temperature-related decreases in stiffness and strength have also been reported for PA12 and PA12-based composites, where the mechanical response at higher temperatures was mainly controlled by the softening of the polymer matrix [25,36,48,49]. In contrast, the increased elongation indicates that the composite material becomes more ductile rather than brittle with increasing temperature. These mechanical parameters will be used to determine the minimum and maximum load levels for fatigue testing and subsequent environmental simulations.

### 3.2. Fatigue Tests

**Figure 4:**
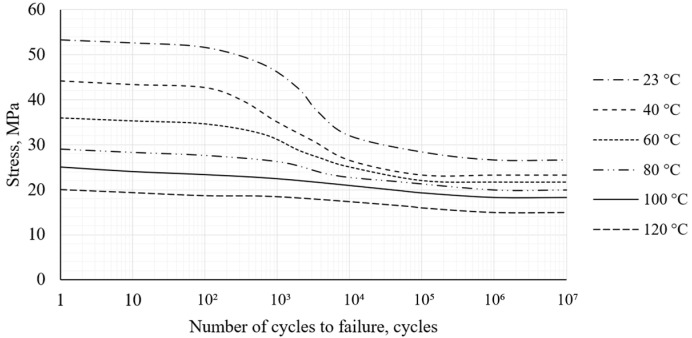
S-N curves for PA12 CF15 specimens.

Fatigue tests were performed under controlled sinusoidal loading with load ratio *R* = 0.1 and frequency 2 Hz. Stress levels were selected within the range of 53.3 MPa to 20 MPa at temperatures between 23 °C and 120 °C. During the tests, the number of cycles to failure was recorded. The specimens reached run-out at 10^6^ cycles (under the tested conditions), with no failure observed. A summary of the fatigue tests results is presented in Figure 4.

The stress-number of cycles curves (S-N curves) show that the maximum load capacity decreases with increasing temperature. At 80–90% or higher load, the specimens fail after a relatively low number of cycles, regardless of the test temperature. At lower load levels, the fatigue resistance increases significantly. At higher temperatures, the mechanical properties of PA12 CF15 become more ductile, which reduces the crack progression rate. On the basis of the fatigue test results, it can be inferred that the 3D-printed PA12 CF15 composite material is appropriate for cyclic loading applications in which the applied load does not exceed approximately 60–70% of the ultimate tensile load, including under elevated temperatures up to 120 °C.

### 3.3. DMA Tests

**Figure 5:**
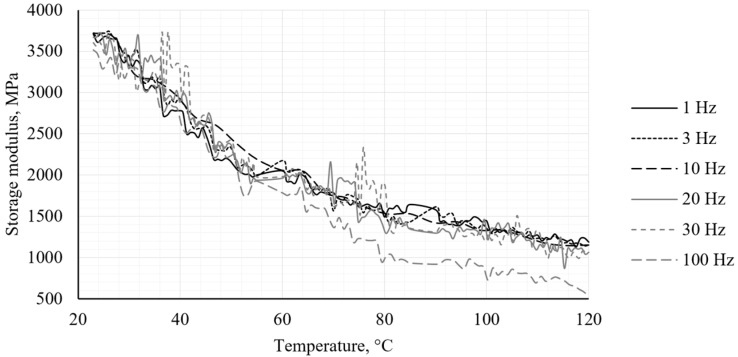
Storage modulus obtained from DMA analysis.

**Figure 6:**
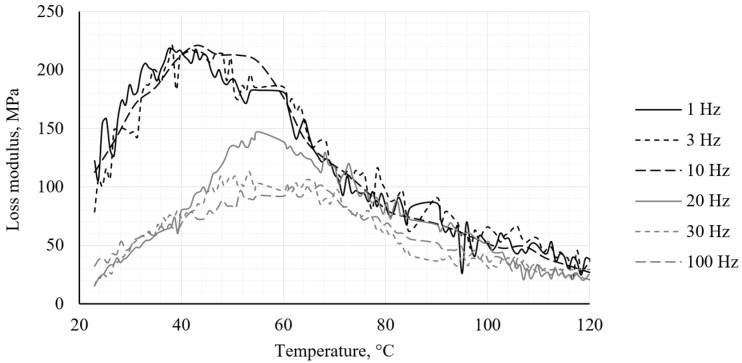
Loss modulus obtained from DMA analysis.

**Figure 7:**
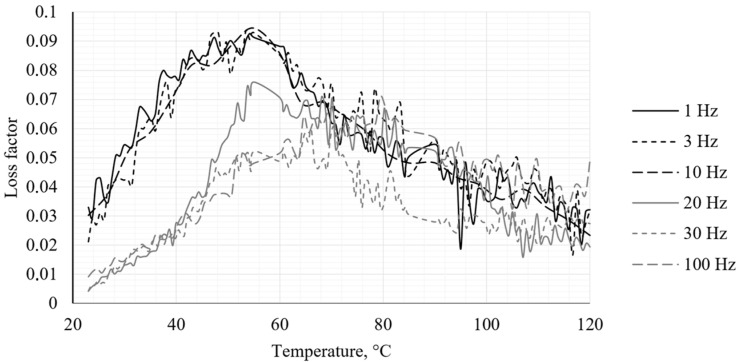
Loss factor obtained from DMA analysis.

The DMA test revealed behavior characteristic of the PA12 CF15 composite materials. The obtained results illustrate the temperature dependence of the storage modulus (*E*′), the loss modulus (*E*″) and the loss factor (*tan δ*) for the specimens, respectively, in Figure 5, Figure 6 and Figure 7.

The storage modulus (*E*′) decreases progressively with increasing temperature, indicating a reduction in the stiffness of the material due to thermal softening of the PA12 matrix. The presence of carbon fiber reinforcement contributes to an increased stiffness at lower temperatures. However, its reinforcing effect diminishes at elevated temperatures as the matrix mobility increases. In comparison with other tests, the storage modulus variation is very similar to similar tests with different additives [50]. The incorporation of carbon fiber results in a substantially higher storage modulus compared to that obtained with alternative reinforcing additives.

The loss modulus (*E*″) increases significantly in the glass transition region, indicating increased molecular mobility and energy dissipation within the polymer matrix. Analysis of the graph indicates that for frequencies above 20 Hz, the excitation frequency exceeds the molecular relaxation rate, resulting in a reduction in the energy dissipated by the system.

The loss factor (*tan δ*) curves show a peak at approximately 55 °C, which is attributed to the glass transition temperature (*T_g_*) of the PA12 CF15 composite at 1 Hz. As the deformation frequency increases, the molecular chains in the composite cannot respond within the imposed time scale, thereby requiring higher temperatures to initiate segmental motion. At an excitation frequency of 100 Hz, the glass transition temperature is estimated to be approximately 65 °C. The DMA results demonstrate that PA12 CF15 exhibits temperature-dependent viscoelastic behavior typical of fiber-reinforced thermoplastics. Below *T_g_*, the material behaves as a stiff elastic composite, while above *T_g_* a significant reduction in stiffness and an increase in damping capacity are observed.

### 3.4. SEM

**Figure 8:**
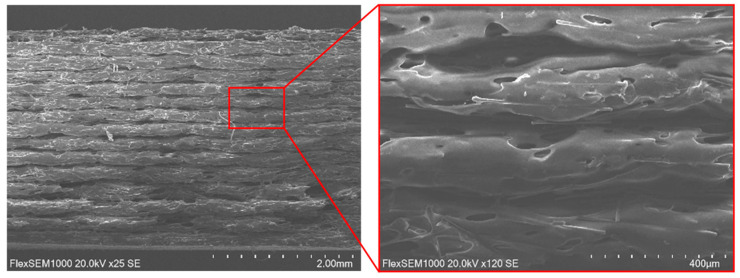
Side view of the PA12 CF15 specimen before deformations.

**Figure 9:**
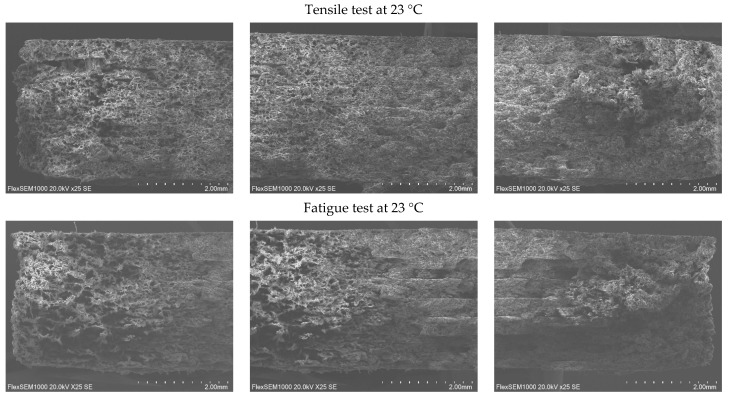
Cross-sectional view of PA12 CF15 specimens after deformations at 23 °C.

**Figure 10:**
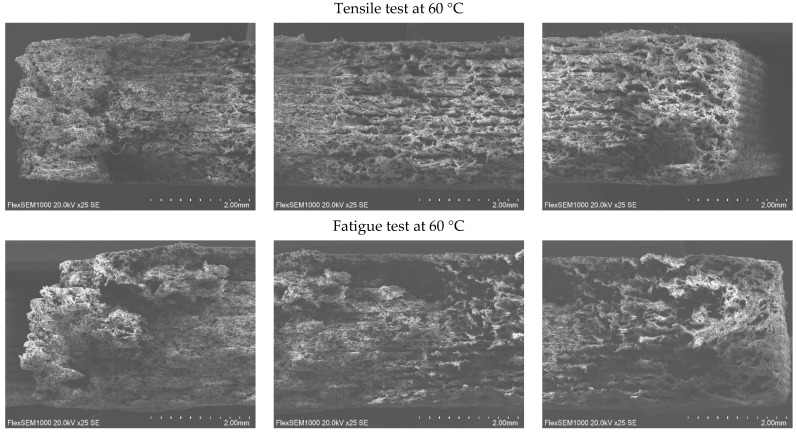
Cross-sectional view of PA12 CF15 specimens after deformations at 60 °C.

**Figure 11:**
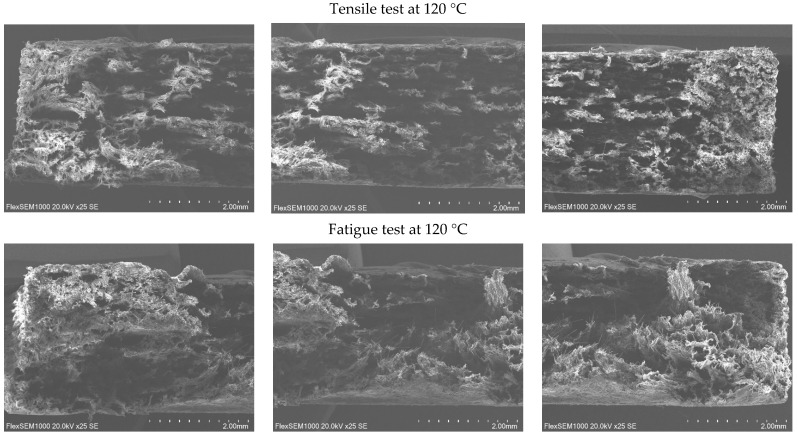
Cross-sectional view of PA12 CF15 specimens after deformations at 120 °C.

The fracture surfaces of the PA12 CF15 composite specimens obtained after mechanical testing at different temperatures were examined by SEM. This study aims to investigate the fracture mechanisms and the influence of temperature on the characteristics of microstructural damage. SEM images of specimens before and after tensile and fatigue tests at different temperatures are presented in Figure 8, Figure 9, Figure 10 and Figure 11.

SEM observations reveal that the carbon fibers exhibit diameters in the range of approximately 8–10 µm.

At 23 °C temperatures, the SEM images reveal relatively smoother fracture surfaces with limited plastic deformation of the polymer matrix. The fracture morphology is characterized by clean fiber breakage and a relatively low number of pulled-out carbon fiber filaments, suggesting a more brittle fracture behavior dominated by matrix cracking and fiber fracture. The interfacial adhesion between the PA12 matrix and the carbon fibers appears sufficient to transfer load; however, limited matrix ductility restricts energy dissipation during fracture.

As the test temperature increases (60 °C), a change in fracture morphology is observed. The fracture surfaces become progressively rougher, accompanied by an increased number of pulled carbon fiber filaments and elongated fiber traces. This behavior indicates a better plastic deformation of the PA12 matrix and a higher interfacial bonding between the matrix and the carbon fibers. The observation of matrix fibrillation and fiber pulling indicates an increase in energy absorption during fracture, evidencing a transition to a more ductile fracture mechanism.

At 120 °C, the matrix deforms and stretches even more. The polymer matrix shows signs of significant deformation before the ultimate failure, which facilitates fiber pullout rather than sudden fracture.

In general, the fracture surfaces of fatigue-tested PA12 CF15 specimens exhibit higher roughness compared to those obtained under quasi-static fracture conditions. This increased roughness is associated with accumulation of damage during cyclic loading, which promotes progressive plastic deformation of the matrix, fiber-matrix interfacial debonding, and the initiation and propagation of microcracks. In summary, the SEM analysis showed that increasing the testing temperature promotes a more fracture behavior in PA12 CF15 composites. The increased surface roughness of the fracture and the higher amount of extracted carbon fiber filaments are consistent with the observed mechanical response, indicating an increased plasticity of the material at elevated temperatures. These findings highlight the strong correlation between temperature-dependent mechanical properties and the underlying fracture mechanisms of the PA12 and carbon fiber composite system.

## 4. Case Study Analysis

### 4.1. Dynamic and Thermal System Analysis

The case study was conducted to characterize the dynamic and thermal behavior of the analyzed system by identifying its resonant frequencies, acceleration amplitudes, and operating temperatures. The experimentally obtained resonant frequencies and acceleration amplitudes were subsequently used as input parameters for finite element analysis to determine the resulting mechanical loads acting on the analyzed model of the intake air manifold. Temperature measurements were performed to identify the maximum operating temperature generated by the engine. These temperature values were incorporated into the mathematical model to calculate and evaluate the actual mechanical properties of the PA12 CF15 composite under realistic operating conditions.

**Figure 12:**
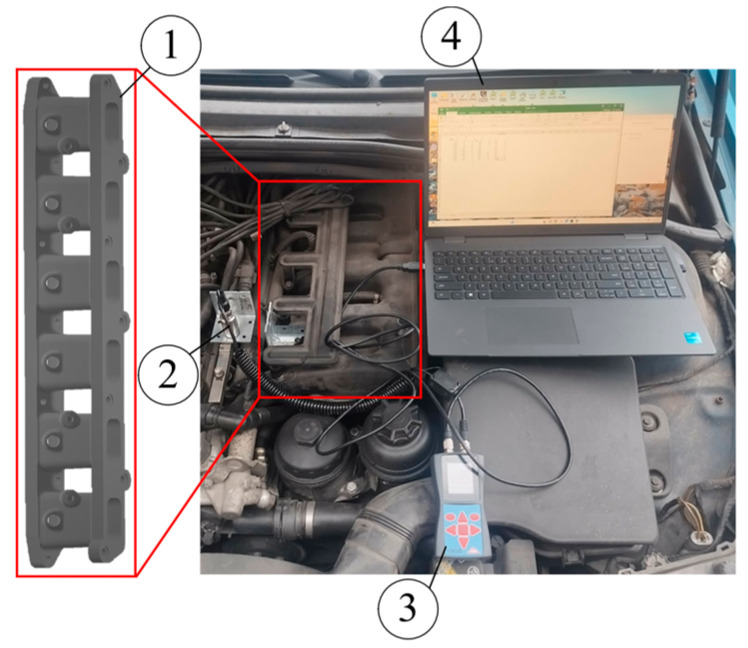
Experimental setup for vibration testing of the designed intake manifold: 1—an intake air manifold; 2—a sensor connected to the engine; 3—a VM25 vibration meter; 4—a computer.

To evaluate the suitability of the PA12 CF15 composite material for the manufacture of an intake air manifold for a gasoline internal combustion engine, a vibration analysis was performed. Under real operating conditions, the intake manifold was mounted directly on the engine block, while additional components, including the intake distributor (2.02 kg) and the throttle body (1.16 kg), were attached to the opposite side. As a result, the manifold was continuously exposed to engine-induced vibrations, dynamic accelerations, and inertial loads, which may significantly affect its structural performance and durability. The experimental setup for the vibration testing of the designed intake manifold is presented in Figure 12.

The experimental study was designed to investigate the resonant frequencies generated by the engine and the corresponding acceleration levels in three orthogonal directions (X, Y, and Z). Measurements were performed using a VM25 vibration meter (Metra Meß- und Frequenztechnik Radebeul GmbH & Co. KG, Radebeul, Germany), with a BMW gasoline engine M54B25 (BMW Group, Munich, Germany). The measured resonant frequency and acceleration data are summarized in Table 4.

**Figure 13:**
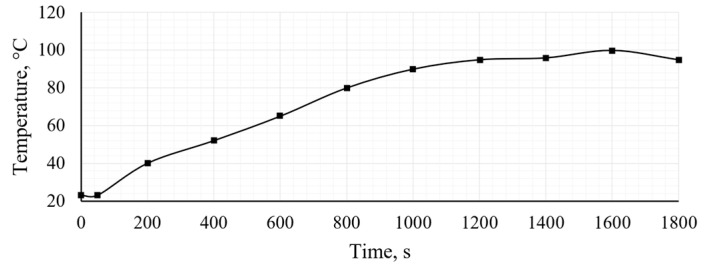
The temperature of engine coolant.

In addition to vibration measurements, the engine operating temperature was continuously monitored and recorded. The obtained temperature-time graph provides important information on the thermal loading conditions to which the intake manifold was subjected during engine operation. The temperature graph is shown in Figure 13.

The experimentally obtained resonance frequencies, acceleration amplitudes, and temperature data served as critical input parameters for the finite element analysis. These data were used to develop a realistic numerical model of the intake manifold, which allowed simulation of coupled thermo-mechanical and dynamic loading conditions.

### 4.2. Finite Element Analysis

Finite element analysis was performed to evaluate the suitability of the PA12 CF15 composite material for automotive components exposed to elevated temperatures. This study investigates the fatigue behavior and dynamic response of a PA12 CF15 composite intake manifold under representative operating conditions of a gasoline internal combustion engine. Numerical simulations were performed using the COMSOL Multiphysics^®^ 6.4 (COMSOL AB, Stockholm, Sweden) program, incorporating experimental data from tensile, fatigue, resonant frequencies, acceleration amplitudes, and operating temperature tests to ensure realistic material modeling and loading conditions. Young’s modulus, Poisson’s ratio, initial yield stress, and isotropic hardening modulus were obtained from tensile tests, while fatigue properties were defined based on experimentally determined S–N curves.

The 3D geometry of the intake manifold was implemented in SolidWorks 2025 (Dassault Systèmes SolidWorks Corp., Waltham, MA, USA) program based on the actual dimensions of the component. Boundary conditions were defined to replicate real mounting conditions, with the manifold rigidly constrained at the interface with the engine block. The masses of the attached components—the intake distributor (2.02 kg) and the throttle body (1.16 kg)—were included in the model as boundary loads defined by analytical functions. The maximum force was assumed to occur at an engine speed of 3500 RPM, corresponding to the maximum acceleration. Based on Newton’s second law, analytical force functions were derived for different spatial directions:

*F* = *mg* + *ma*
*sin* (2 *π f t*), (1)

where *m* is the mass, *g* is the acceleration of free fall, *a* is the maximum acceleration, *f* is the resonant frequency and *t* is the time.

*F_cycX_* = 3.18 ∙ 5 ∙ *sin* (2*π* ∙ 52 ∙ *t*), (2)

*F_cycY_* = 3.18 ∙ 13 ∙ *sin* (2*π* ∙ 162 ∙ *t*), (3)

*F_cycZ_* = 3.18 ∙ 9.822 + 3.18 ∙ 4 ∙ *sin* (2*π* ∙ 336 ∙ *t*), (4)

**Figure 14:**
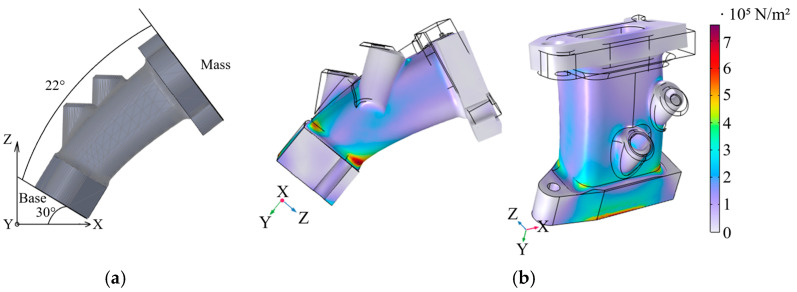
Finite element analysis: (**a**) Indication of force directions, base, and mass in mathematical model; (**b**) Von Mises stress distribution.

The designed geometry was simplified to optimize the computational efficiency of numerical analysis. Consequently, the applied loads were recalculated to account for the reduced geometry and equivalent force magnitudes were applied to the analyzed intake in the respective coordinate directions to ensure consistency with the original loading conditions. Temperature-dependent material behavior was taken into account by incorporating engine operating temperature data measured during vibration testing, enabling the adjustment of material stiffness and strength under thermal loading conditions. Since the 3D-printed model is not homogeneous, the raster angle was selected to align with the direction of the highest expected stresses. Although this approach does not explicitly account for fiber orientation, fiber–matrix interfacial effects, or internal defects, it provides a computationally efficient and sufficiently accurate representation for global structural and fatigue assessments. The results were subsequently evaluated qualitatively to assess stress distribution and magnitude. The mathematical model of the intake manifold is presented in Figure 14a.

After performing finite element calculations, the stress–strain state of the intake manifold structure was evaluated under cyclic dynamic loading and elevated operating temperatures. The numerical results indicate that the maximum strains and displacements are concentrated in the mounting regions and at the transitions between the flange and the air duct, where the highest load concentrations occur. The obtained first principal strain values remain below the critical limits of the PA12 CF15 material determined from experimental testing. The direction of the force caused by vibrations is shown in Figure 14b and is associated with the results of the vibration measurements.

The mesh convergence study confirmed that the numerical results stabilize when a Fine mesh is applied. Variations in the first principal strain and displacement magnitude between the Fine, Finer, and Extra fine meshes are negligible, indicating mesh independence. Consequently, the Fine mesh was selected for further analysis because it provides a suitable compromise between computational accuracy and efficiency. The calculation results are presented in Table 5.

**Figure 15:**
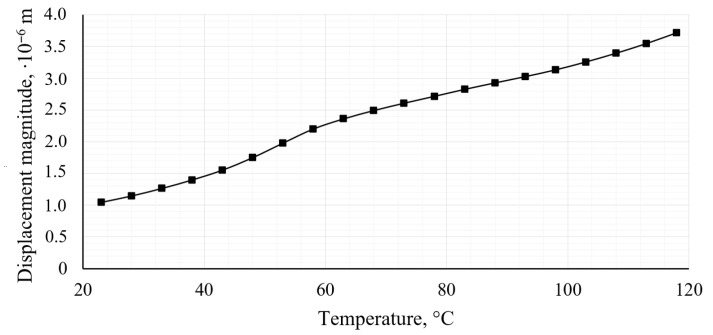
Displacement magnitude at different temperatures.

Thermal-mechanical analysis revealed a clear increase in strain and displacement with increasing operating temperature in the range of 23 °C to 120 °C. The magnitude of displacement at different temperatures is shown in Figure 15.

The maximum value of the first principal strain value increased from 7.66 × 10^−5^ to 2.72 × 10^−4^, while the magnitude of displacement increased from 1.05 × 10^−5^ m to 3.72 × 10^−5^ m. This behavior is primarily attributed to the temperature-dependent reduction in material stiffness, as the elastic modulus of the PA12 CF15 composite decreases with increasing temperature. Despite the observed increase, the deformation levels at the highest temperature considered do not approach the threshold values associated with plastic deformation or premature fatigue failure.

Fatigue evaluation showed that the intake manifold can withstand the analyzed cyclic loading conditions, with no plastic deformations at low loads. These results confirm that the chosen raster angle and structural design are appropriate to ensure overall stiffness and fatigue life, despite the fact that local microstructure effects such as fiber orientation, porosity, and fiber-matrix interface behavior were not explicitly modeled. In general, the finite element analysis confirms that PA12/CF15 exhibits sufficient mechanical strength, fatigue resistance, and dynamic stability for application in intake air manifolds of gasoline engines, supporting its suitability for use in vibration-intensive automotive environments.

## 5. Conclusions

In this work, experimental and numerical studies on the thermomechanical properties, fatigue behavior, and engineering application case study of a 3D-printed PA12 CF15 composite were presented. Based on quasi-static tensile testing, fatigue experiments, DMA, SEM, vibration measurements, and finite element simulations, the following conclusions could be drawn:

1. Quasi-static tensile tests showed a decrease in elastic modulus, yield strength, and ultimate tensile strength with increasing temperature in the range of 23 °C to 120 °C. This behavior was attributed to the increased mobility of the PA12 matrix at elevated temperatures, which progressively reduced the reinforcement efficiency of the carbon fibers. At the same time, the elongation at break increased, indicating a transition from a more brittle response at 23 °C temperature to a plastic deformation at higher temperatures.
2. Fatigue tests showed that the fatigue resistance of PA12 CF15 was highly dependent on temperature and load. As the load increased, the durability and maximum number of cycles of the material decreased, and as the test temperature increased, the composite became more plastic, resulting in a different fatigue effect with increasing temperature. Regardless of temperature, fatigue occurred if the load amplitude was greater than 80% of the maximum tensile force. However, at moderate loads (≤60–70% of maximum tensile strength) and temperatures above 80 °C, no failure was observed within the investigated number of fatigue cycles (10^6^ cycles). These results indicated that FFF-printed PA12 CF15 is suitable for cyclic loading conditions, where appropriate safety margins apply.
3. The DMA showed a continuous decrease in the storage modulus with increasing temperature. Based on the loss modulus and the loss coefficient, the glass transition temperature was obtained at 55 °C. As the excitation frequency increased, the glass transition temperature changed and reached 65 °C. The carbon fiber reinforcement significantly improved the stiffness of the composite below the glass transition temperatures.
4. The SEM analysis provided information on the change in fracture morphology with increasing temperature. At 23 °C, the fracture surfaces were relatively smooth and dominated by cracking of the matrix and fiber breakage, indicating a brittle failure mechanism. At higher temperatures, increased matrix plastic deformation, fiber pull-out, and interfacial debonding were observed, corresponding to a more plastic fracture behavior.
5. Experimental vibration measurements performed on a gasoline internal combustion engine provided realistic acceleration amplitudes and resonance frequencies acting on the intake manifold. These data revealed that the component was subjected to multi-directional dynamic loading combined with elevated operating temperatures, highlighting the necessity of evaluating both fatigue and thermo-mechanical behavior for additively manufactured automotive components.
6. Finite element simulations incorporating experimentally derived temperature-dependent material properties and measured vibration loads demonstrated that the PA12 CF15 intake manifold remained within elastic deformation limits under the operating conditions analyzed. The highest strains and displacements were located in the mounting regions and geometric transitions but remained well below the critical values associated with plastic deformation or fatigue failure.

The combined experimental-numerical approach provided a preliminary indication that the 3D-printed PA12 CF15 exhibits sufficient stiffness, fatigue resistance, and dynamic stability for use in devibration-intensive automotive parts or in other thermo-mechanical applications, provided that the orientation of the print, load levels, and operating temperatures are properly considered. The results offer valuable design-relevant data and demonstrate that additive manufacturing can be a viable alternative to conventional manufacturing methods for functional intake air system components or for other engineering applications.

## Figures and Tables

**Table 1:** Basic properties of analyzed polymer PA12 CF15 (provided by the manufacturer) [32].

Properties	Value	Standard
Density, g/cm^3^	1.07	-
Tensile strength, MPa	120	ISO 527 [33]
Tensile modulus, MPa	7300	
Elongation, %	5	
Charpy impact strength (notched) at 23 °C, kJ/m^2^	15	ISO 179 [34]
Charpy impact strength (unnotched) at 23 °C, kJ/m^2^	75	
Melting point, °C	178	ISO 3146 [35]

**Table 2:** The printing parameters of the specimen.

Parameters	Value
Temperature of the nozzle, °C	265
Temperature of the heated bed, °C	100
Weight of the specimen, g	6.42
Printing speed of the outer wall, mm/s	60
Printing speed of the inner wall, mm/s	60
Cooling, %	100
Height of the layer, mm	0.2
Raster angle, in degrees	45
Infill, %	100
The number of contours	5
Diameter of the nozzle, mm	0.6

**Table 3:** Mechanical parameters of PA12 CF15 (calculated from the tensile tests).

Parameters	Values					
Testing temperature, °C	23	40	60	80	100	120
Elastic modulus, GPa ± SD	2.18 ± 0.18	1.56 ± 0.13	1.00 ± 0.08	0.82 ± 0.02	0.72 ± 0.01	0.60 ± 0.02
Yield strength, MPa ± SD	21.59 ± 1.14	14.61 ± 1.12	12.29 ± 1.28	9.05 ± 1.32	7.52 ± 1.48	6.51 ± 1.45
Ultimate tensile strength, MPa ± SD	49.07 ± 0.88	40.31 ± 0.72	32.70 ± 1.18	26.60 ± 1.15	22.78 ± 0.97	18.68 ± 0.91
Tangent modulus, MPa ± SD	266.01 ± 3.56	248.95 ± 2.98	220.07 ± 2.83	176.36 ± 2.35	144.40 ± 2.12	117.10 ± 2.26
Elongation at break, % ± SD	8.71 ± 1.06	9.40 ± 1.18	12.73 ± 1.01	12.49 ± 1.26	12.49 ± 1.69	19.04 ± 1.89

**Table 4:** The data of resonant frequency and acceleration.

	X Direction		Y Direction		Z Direction	
Revolutions per Minute (RPM)	Resonant Frequency, Hz	Acceleration, m/s^2^	Resonant Frequency, Hz	Acceleration, m/s^2^	Resonant Frequency, Hz	Acceleration, m/s^2^
750	36	1	182	0	180	0
1000	57	1	160	0	300	0
1500	72	0.5	188	1	365	0
2000	101	2	156	1	370	2
2500	625	2	220	1	370	1
3000	466	4	144	10	382	2
3500	52	5	162	13	336	4
4000	504	6	185	5	307	3

**Table 5:** The mesh reliability analysis.

	Extra Fine	Finer	Fine	Normal	Coarse	Coarser
The number of domain elements	476,516	165,942	63,301	29,767	15,347	8301
The number of boundary elements	83,614	36,465	18,008	10,559	6658	4097
Value of the first principal strain	3.7438 × 10^−4^	3.7443 × 10^−4^	3.7391 × 10^−4^	3.8033 × 10^−4^	3.7615 × 10^−4^	3.8369 × 10^−4^
Displacement magnitude, m	5.1702 × 10^−5^	5.1703 × 10^−5^	5.1701 × 10^−5^	5.1643 × 10^−5^	5.1517 × 10^−5^	5.1291 × 10^−5^

## Data Availability

The original contributions presented in this study are included in the article. Further inquiries can be directed to the corresponding author.
